# Aquaglyceroporin Modulators as Emergent Pharmacological Molecules for Human Diseases

**DOI:** 10.3389/fmolb.2022.845237

**Published:** 2022-02-03

**Authors:** Catarina Pimpão, Darren Wragg, Inês V. da Silva, Angela Casini, Graça Soveral

**Affiliations:** ^1^ Research Institute for Medicines (iMed.ULisboa), Faculty of Pharmacy, Universidade de Lisboa, Lisboa, Portugal; ^2^ Department of Pharmaceutical Sciences and Medicines, Faculty of Pharmacy, Universidade de Lisboa, Lisboa, Portugal; ^3^ Department of Chemistry, Technical University of Munich, Munich, Germany

**Keywords:** aquaporin, glycerol permeability, functional assays, computational methods, regulation, expression modulators, inhibitors

## Abstract

Aquaglyceroporins, a sub-class of aquaporins that facilitate the diffusion of water, glycerol and other small uncharged solutes across cell membranes, have been recognized for their important role in human physiology and their involvement in multiple disorders, mostly related to disturbed energy homeostasis. Aquaglyceroporins dysfunction in a variety of pathological conditions highlighted their targeting as novel therapeutic strategies, boosting the search for potent and selective modulators with pharmacological properties. The identification of selective inhibitors with potential clinical applications has been challenging, relying on accurate assays to measure membrane glycerol permeability and validate effective functional blockers. Additionally, biologicals such as hormones and natural compounds have been revealed as alternative strategies to modulate aquaglyceroporins *via* their gene and protein expression. This review summarizes the current knowledge of aquaglyceroporins’ involvement in several pathologies and the experimental approaches used to evaluate glycerol permeability and aquaglyceroporin modulation. In addition, we provide an update on aquaglyceroporins modulators reported to impact disease, unveiling aquaglyceroporin pharmacological targeting as a promising approach for innovative therapeutics.

## Introduction

Aquaporins (AQPs) are transmembrane protein channels found in all life forms, including archaea, eubacteria, fungi, and plants (Zardoya, 2005). AQPs facilitate the bidirectional transport of water and small polar solutes (such as glycerol) across cell membranes, triggered by osmotic or solute gradients (Verkman, 2012). In humans, the 13 AQPs (AQP0-12) identified so far are widely distributed in the body and found in many tissues and cell types. According to their pore selectivity, AQPs are grouped in 1) orthodox aquaporins (AQP0, AQP1, AQP2, AQP4, AQP5, AQP6 and AQP8) that are considered primary selective to water, 2) aquaglyceroporins (AQP3, AQP7, AQP9 and AQP10) that in addition to water, also transport glycerol and other small neutral solutes; and 3) subcellular or unorthodox aquaporins (AQP11 and AQP12) with distinct evolutionary pathway, localizing in intracellular membranes (Ishibashi et al., 2014) and with uncertain permeability (Ishibashi et al., 2021), although AQP11 was shown as a water and glycerol channel (Madeira et al., 2014a). A few members can also transport ammonia (ammoniaporins) (Jahn et al., 2004), hydrogen peroxide (peroxiporins) (Rodrigues et al., 2016; Prata et al., 2019; Rodrigues et al., 2019; Bestetti et al., 2020), and gases such as CO_2_, O_2_ (Geyer et al., 2013) or NO (Herrera et al., 2006), among other solutes (Wu and Beitz, 2007). Aquaporin research has gained much interest in recent years due to their physiological importance and implication in a broad range of disorders (Soveral et al., 2016), prompting the discover of AQP-modulators for potential therapeutic applications (Soveral and Casini, 2017). In particular, the aquaglyceroporin sub-family has attracted the attention of the research community due to their important roles in health and disease, being revealed as crucial players in a number of metabolic and inflammatory disorders (Madeira et al., 2015; da Silva and Soveral, 2017; da Silva et al., 2018a; Calamita et al., 2018; da Silva et al., 2018b; Mendez-Gimenez et al., 2018; da Silva et al., 2020a), including infectious responses (Tesse et al., 2021), inflammasome activation (da Silva et al., 2020b) and migration and survival of immune cells (da Silva and Soveral, 2021).

This review summarizes the current knowledge of aquaglyceroporins involvement in pathophysiological conditions and describe the experimental approaches to evaluate glycerol permeability and aquaglyceroporin modulation. In addition, we provide an update on aquaglyceroporins modulators reported to impact in disease, revealing aquaglyceroporins’ potential as drug targets for new therapies against pathologies with dysregulated aquaglyceroporin expression.

## Pathophysiological Roles of Mammalian Aquaglyceroporins

The aquaglyceroporins (AQP3, AQP7, AQP9, and AQP10) are involved in a series of malfunctions and diseases, most of them related to their ability to facilitate glycerol diffusion across cell membranes, a feature that is determined by a larger constriction region of their size selectivity filter compared to orthodox aquaporins (pore diameter of about 2.8 Å and 3.5 Å in human orthodox and aquaglyceroporins, respectively) (Hub and de Groot, 2008).

**FIGURE 1:**
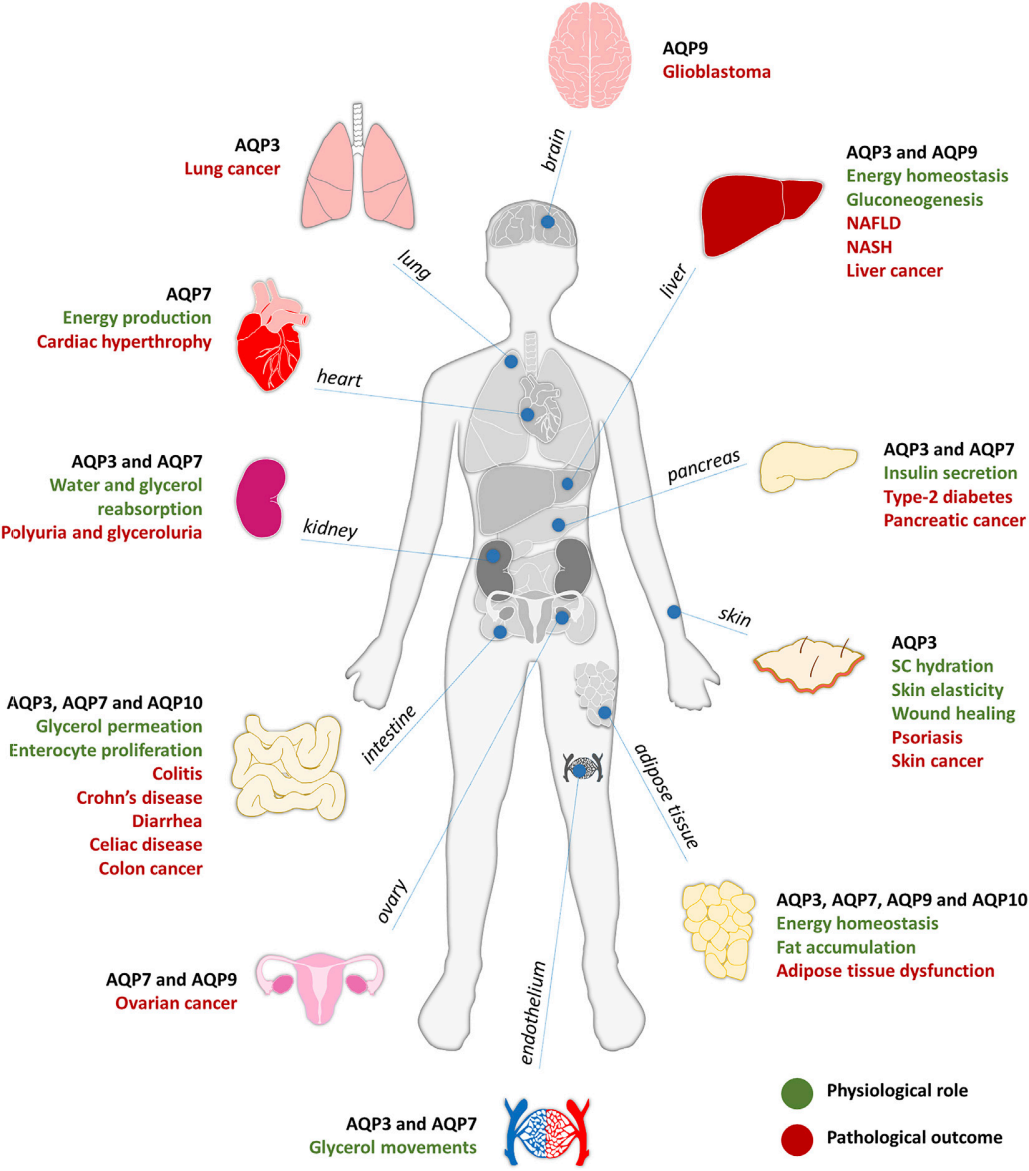
Aquaglyceroporins involvement in physiology (in green) and pathology (in red), described for each organ of the human body.

Glycerol is an important molecule in energy metabolism for its crucial involvement in lipogenesis in feeding conditions and lipolysis and gluconeogenesis during fasting, contributing for energy homeostasis (Rodriguez et al., 2015a). Due to their ability to transport glycerol bridging tissues from different organs, aquaglyceroporins are key players in many physiological conditions and their dysfunction is related to disease (Soveral et al., 2016), as depicted in Figure 1. Increasing evidence show their involvement in a broad spectrum of pathophysiological conditions, such as skin dehydration, obesity, fatty liver, diabetes, intestinal disorders, cardiovascular disease and cancer, among others, highlighting the great pharmacological potential of these transmembrane channels (Soveral et al., 2016).

Skin represents the first line of defense of the human body covering the entire external body surface, where aquaglyceroporins are essential for skin physiology (da Silva et al., 2021). AQP3 is found in the basal layer of epidermal keratinocytes where it plays an important role in epidermal and stratum corneum (SC) hydration, since glycerol acts as a humectant that preserves the skin moisture (Verkman, 2009). AQP3-deficient mice show reduced SC hydration, accompanied with impaired skin elasticity and delayed wound healing (Hara-Chikuma and Verkman, 2006; Hara-Chikuma and Verkman, 2008a). This outcome results from the reduction of glycerol permeation, followed by decreased SC and epidermis glycerol content (Hara et al., 2002; Verkman, 2009). Glycerol administration can correct these skin defects, overcoming the absence of this aquaglyceroporin in epidermal keratinocytes (Hara and Verkman, 2003; Hara-Chikuma and Verkman, 2008b; Verkman, 2009). Moreover, when AQP3 is overexpressed, epidermal proliferation is enhanced due to the higher AQP3-mediated glycerol permeability and can result in hyperproliferative skin disorders such as psoriasis and skin tumors (da Silva et al., 2021; Hara-Chikuma and Verkman, 2008a; Verkman et al., 2014).

Adipose tissue and liver are the main organs involved in body energy homeostasis. AQP7 is expressed in both adipocyte and capillary plasma membranes of human adipose tissue (Skowronski et al., 2007; Laforenza et al., 2013) while AQP9 is localized in the plasma membrane of adipocytes and hepatocytes, being both key regulators of fat accumulation and glucose homeostasis (Maeda et al., 2009; Madeira et al., 2015). For this reason, they have been associated with metabolic disorders such as obesity and metabolic syndrome (da Silva and Soveral, 2017; da Silva et al., 2018b). Although other aquaglyceroporins have also been detected in human adipocytes (Rodriguez et al., 2011; Laforenza et al., 2013; Madeira et al., 2014a), AQP7 is the main glycerol channel responsible for the efflux and uptake of glycerol from adipocytes (Marrades et al., 2006; Maeda, 2012). During fasting or exercise, AQP7 gene expression is increased, whereas in the feeding state, AQP7 is downregulated, with expression inversely related with plasma insulin levels (Kishida et al., 2000). AQP7 knockout mice reveal adipocyte hypertrophy and increased adipocyte mass due to the reduced glycerol permeability of the plasma membrane and increased glycerol kinase activity, resulting in intracellular triglycerides accumulation (Hara-Chikuma et al., 2005; Hibuse et al., 2005; Rodriguez et al., 2006). Adipocyte lipid storage above normal levels and associated lipotoxicity can lead to inflammation, insulin resistance and obesity (da Silva and Soveral, 2017; Marrades et al., 2006), a cluster of disorders representing the metabolic syndrome (da Silva et al., 2018b). Furthermore, in a model of obese rats with type 2 diabetes (Kishida et al., 2000; Lee et al., 2005), AQP7 mRNA expression in adipose tissue was upregulated, possibly contributing to an augmented glycerol input for hepatic gluconeogenesis and subsequent hyperglycemia (Rojek et al., 2008). Interestingly, AQP7 is regulated in response to physical training in a gender-dependent manner, with women adipose tissue showing higher abundance when compared to men (Lebeck et al., 2012). AQP10 was also found expressed in human adipocytes and was proposed to represent an alternative pathway to AQP7 for glycerol efflux from adipose tissue (Laforenza et al., 2013), stimulated by the decreased pH observed during lipolysis (Gotfryd et al., 2018), assuring the maintenance of normal or low glycerol contents inside the adipocyte and protecting humans from obesity.

In the endocrine pancreas, AQP7 is the main aquaglyceroporin and is responsible for glycerol uptake into β-cells (Delporte et al., 2009). AQP7 knockout mice showed increased β-cell glycerol and triglycerides content and augmented glycerol kinase activity, associated with increased pancreatic insulin mRNA levels and insulin secretion at an elevated rate (Matsumura et al., 2007). These findings suggest that AQP7 is a key player in the regulation of insulin biosynthesis and secretion, indirectly controlling intracellular glycerol content and triglycerides synthesis (Rodriguez et al., 2011; Mendez-Gimenez et al., 2018). More recently, AQP7 was found to modulate inflammatory phenotype of endocrine pancreatic beta-cells, and to play a role in β-cells proliferation, adhesion, and migration (da Silva et al., 2020a).

In the liver, AQP9 was identified as the major route for glycerol uptake where it can be used in gluconeogenesis (Rojek et al., 2007; Jelen et al., 2011). AQP9-null obese mice with type 2 diabetes showed a significant increase in plasma glycerol levels accompanied by low fasting glucose levels. These findings suggest an impairment in glucose production due to AQP9 deficiency, limiting hepatic glycerol uptake for gluconeogenesis and highlighting AQP9 as a crucial player in both glycerol and glucose metabolism (Rojek et al., 2007; Maeda et al., 2009; Jelen et al., 2011; Rodriguez et al., 2011). Additionally, in a model of non-alcoholic fatty liver disease (NAFLD), AQP9 overexpression resulted in increased intracellular triglycerides, free fatty acids and glycerol levels, leading to the aggravation of steatosis, and this could be reversed with AQP9 suppression (Wang et al., 2013). Recently, hepatic AQP9 was found downregulated in obese patients with NAFLD and steatohepatitis (NASH), with even lower levels in patients with insulin-resistance, suggesting a compensatory mechanism to prevent glycerol availability for triglycerides accumulation and hepatic gluconeogenesis (Rodriguez et al., 2014). Interestingly, although hepatic AQP9 protein level was similar in obese male and female patients, the lower glycerol permeability detected in obese women hepatocytes might suggest a decreased risk of developing insulin resistance and NAFLD (Rodriguez et al., 2014; Rodriguez et al., 2015b).

In the heart, AQP7 has an important role in energy production (Hibuse et al., 2005). AQP7 knockout mice exhibited lower cardiac glycerol and ATP contents, with development of cardiac hypertrophy and higher mortality when cardiac muscle is in conditions of pressure overload (Hibuse et al., 2009). It has been recently proposed that AQP7-mediated glycerol metabolism plays a key role preventing myocardial ischemia-related damage (Ishihama et al., 2021).

In endothelium, AQP3 was identified as the predominant aquaglyceroporin, being responsible for endothelial glycerol permeation (da Silva et al., 2018a). AQP7 was shown to be localized in mice endothelium of both white and brown adipose tissue, heart, and skeletal muscle. In addition, AQP7 expression is upregulated in streptozocin-induced diabetes mellitus, indicating that insulin can regulate adipose tissue function through control of glycerol influx and outflow through the vascular endothelium (Skowronski et al., 2007). AQP7 expression in the endothelium of adipose tissue capillaries and in adipocytes plasma membrane was also confirmed in humans while AQP10 was reported to be localized in the adipocytes and not in the microvessels (Laforenza et al., 2013). Moreover, in the human small intestine, an AQP10 variant was identified in the capillary endothelial cells in small intestine villi (Li et al., 2005).

In intestinal inflammatory disorders, AQP3 is expressed in the basolateral membrane of colonic epithelial cells and was associated with enterocyte proliferation in a AQP3 null mice model of colitis, that showed epithelial cell damage and loss, colonic hemorrhage, impaired enterocyte proliferation and reduced survival compared to wild-type mice (Laforenza, 2012). With oral glycerol administration, these symptoms were reversed by significantly improving survival and reducing the severity of colitis, revealing the important role of AQP3 in enterocyte proliferation due to its glycerol-transporting function. Thus, AQP3 modulation was suggested with great potential for disorders associated with abnormal enterocyte proliferation, such as Crohn’s disease (Thiagarajah et al., 2007). Additionally, in 2,4,6-trinitrobenzene sulfonic acid (TNBS)-induced rat colitis model, that resembles human Crohn’s Disease, AQP3 and AQP8 mRNA and protein expression were found downregulated, with aggravated intestine inflammation and injury (Zhao et al., 2014). In addition, AQP3, AQP7, AQP10 and AQP11 expression was found strongly decreased in patients with celiac disease, that was recovered by dietary gluten withdrawal (Laforenza et al., 2010). Furthermore, AQP3 and AQP2 were found upregulated in a magnesium sulphate-induced diarrhea mice model which could be reverted with tannin extract treatment that has antidiarrheal properties. Tannin extract downregulates AQP2 and AQP3 expression through the suppression of PKA/pCREB signaling pathway, leading to decreased fecal water content in the colon (Liu et al., 2014).

The kidney is critical for the maintenance of body water homeostasis and the involvement of AQPs in acute and chronic kidney diseases is well recognized (Su et al., 2020). Although AQP2 and AQP1 have a prominent role in urine concentration, AQP3 expression at the basolateral membrane of collecting duct principal cells is increased by thirst and by vasopressin or aldosterone secretion, representing a potential water exit pathway from these cells (Noda et al., 2010). Mice lacking AQP3 show decreased urine concentration ability and polyuria (Ma et al., 2000; Verkman, 2008a; Verkman et al., 2014). AQP7 is localized in the proximal tubule apical membrane, where it facilitates glycerol and water transport (Rojek et al., 2008; Noda et al., 2010). Studies with AQP7 knockout mice showed glyceroluria, suggesting a role of AQP7 in glycerol reabsorption in the proximal straight tubule that cannot be compensated by other aquaglyceroporins (Sohara et al., 2005; Noda et al., 2010).

Increasing evidence show the implication of aquaglyceroporins in cancer and metastasis, where their expression level is in general positively correlated with tumor stage and aggressiveness (Papadopoulos and Saadoun, 2015). AQP3 is the aquaglyceroporin most implicated in tumorigenesis, being aberrantly overexpressed in cancers of different origin, such as skin cancer (Hara-Chikuma and Verkman, 2008c), colon (Zhang et al., 2020), lung (Liu Y. L. et al., 2007), liver (Chen et al., 2018) and pancreatic cancer (Direito et al., 2017). AQP3-facilitated glycerol transport generates ATP and mediates the growth and survival of tumor cells (Verkman et al., 2014) and, additionally, AQP3 ability to permeate H_2_O_2_ may promote tissue inflammation by increasing reactive oxygen species in macrophages (Hara-Chikuma et al., 2020) and contribute to the setting of inflammatory response (da Silva et al., 2020b; da Silva and Soveral, 2021). Targeting AQP3 expression reduces several intracellular signaling pathways, leading to reduced cell proliferation, migration, and invasion (De Ieso and Yool, 2018), suggesting that AQP3 modulators could help in the prevention and therapy of several tumors with abnormal expression of this aquaglyceroporin (Aikman et al., 2018). AQP7 and AQP9 are also highly expressed in malignant ovarian tumor cells comparing to benign tumor or normal tissue, suggesting their role in ovarian carcinogenesis (Yang et al., 2011). Furthermore, AQP9 was also found upregulated in human glioblastoma probably due to its contribution for energy metabolism and its ability of lactate and glycerol clearance from the extracellular space to counteract the lactate acidosis that is associated with this specific type of cancer (Warth et al., 2007; Tan et al., 2008). In hepatocellular carcinoma (HCC), AQP3 was upregulated, and AQP7 and AQP9 were downregulated (Chen et al., 2016), being significantly associated with the aggressive features of HCC. In addition, AQP9 downregulation was also correlated with apoptosis resistance (Jablonski et al., 2007), invasion and epithelial-to-mesenchymal transition (EMT) (Zhang et al., 2016).

Overall, due to their involvement in a broad range of pathophysiological conditions, aquaglyceroporins revealed as promising targets for drug discovery, boosting the search for potent and selective modulators with pharmacological properties.

## Aquaglyceroporin Functional Assays

**FIGURE 2:**
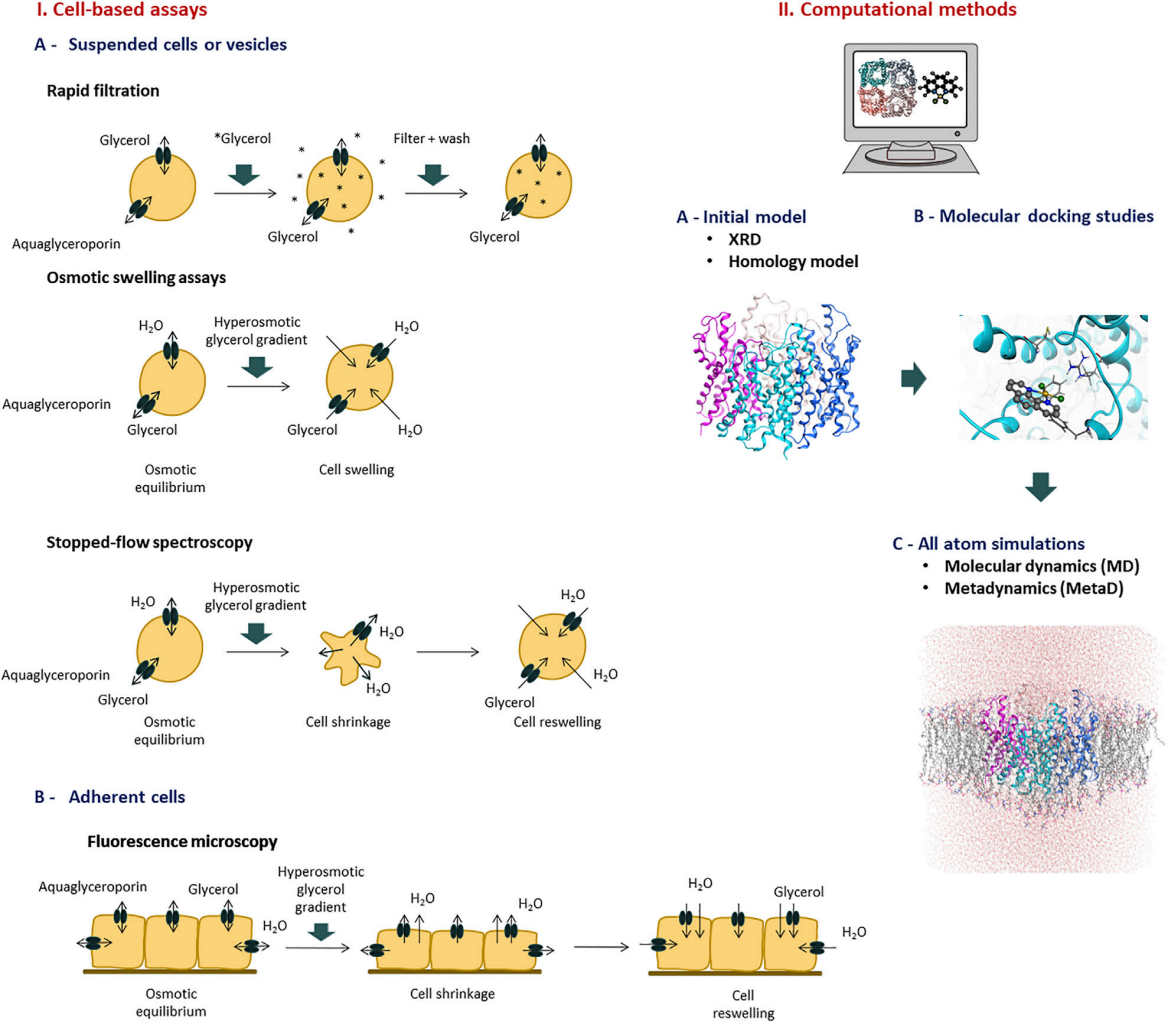
Methods to evaluate aquaglyceroporins function and screen potential modulators. Representative assays of I) Cell based assays, and II) Computational methods.

The identification and validation of aquaglyceroporin modulators (inhibitors) relies on *in vitro* and *in silico* assays to measure glycerol permeability in membranes, providing structure-activity relationships able to validate drug hits (Madeira et al., 2016). The methods currently used to evaluate glycerol permeability and screen potential aquaglyceroporin modulators are summarized in Figure 2.

### Cell-Based Assays

Aquaglyceroporin activity has been detected in procaryotic and eucaryotic cells and isolated membranes from a wide range of organisms, such as bacteria (Froger et al., 2001), yeast (Soveral et al., 2007; Soveral et al., 2008; Mosca et al., 2018), and mammalian cells (da Silva et al., 2018a; da Silva et al., 2020a; Madeira et al., 2013), using whole cells or isolated intracellular vesicles (Noronha et al., 2014). In addition, the functional characterization of procaryotic and eukaryotic aquaporins has been achieved by heterologous expression in oocytes of the frog *Xenopus laevis* (Chauvigne et al., 2021) or in the yeast strain *Saccharomyces cerevisiae* devoid of endogenous aquaporins, where their glycerol transporting ability as well as their mechanisms of gating were uncovered (Bjorkskov et al., 2017; Gotfryd et al., 2018; Mosca et al., 2018; Sabir et al., 2020). The appropriate matching of cell model and functional assay is crucial to obtain accurate information on aquaglyceroporin physiological relevance, identify potential modulators and evaluate their impact in the selected pathophysiological conditions.

#### Rapid Filtration Technique

The rapid filtration technique has generally been used to measure the uptake of solute into cells and isolated membrane vesicles. Uptake of glycerol across the cell membrane is facilitated by aquaglyceroporins, thus measuring the accumulation rate of radiolabeled (usually ^14^C or ^3^H) glycerol provides an experimental approach to determine aquaglyceroporin activity in a variety of cell types and vesicles. Cells are rapidly mixed with an incubation medium containing radioactively labeled permeant solute. After appropriate time intervals, the uptake is blocked by adding stop solution and cells are dropped on a filter that is subsequently washed and counted for radioactivity. Efflux from vesicles pre-loaded with labelled substrates can also be measured using similar procedures. Although these assays are simple and rapid and can provide mechanistic information on transport kinetics (Liu Y. et al., 2007; Ishii et al., 2011; Katano et al., 2014), some limitation include the high costs of radiolabeled glycerol and its disposal and the current safety policies that restrict radioactivity handling and may limit their usage.

#### Osmotic Swelling Assays

The osmotic lysis assay has been largely used for evaluation of aquaglyceroporins modulation in erythrocytes (Campos et al., 2011; Martins et al., 2012). Human erythrocytes express AQP3, and when challenged with hyperosmotic glycerol solutions, cells swell and eventually hemolyze due to glycerol and water influx. The rate of swelling and hemolysis can be monitored spectrophotometrically and used to calculate the glycerol permeability (Liu Y. et al., 2007; Campos et al., 2011; Madeira et al., 2016).

*Xenopus laevis* oocytes have a low intrinsic water permeability and were the first cells used to show AQP1 inhibition by mercury chloride (Preston et al., 1992). Due to their lack of endogenous AQP expression, *Xenopus* oocytes have been considered a good model to study aquaglyceroporin activity and regulation (Beitz et al., 2009; Madeira et al., 2016). Oocytes are microinjected with mRNA encoding aquaglyceroporins and then subjected to an inwardly directed solute gradient, leading to solute and water influx and swelling. The oocyte swelling kinetics is followed by video microscopy where the increase in cell volume is quantified (Beitz et al., 2009). *Xenopus* oocytes swelling assays have revealed limitations due to cell geometry and extracellular unstirred layers artifacts, that may explain the significant variability reported in inhibition studies (Liu et al., 2002; Verkman et al., 2014; Tradtrantip et al., 2017).

#### Fluorescence Microscopy Assays

Fluorescence microscopy can be used to evaluate the permeability of living cells. AQP-expressing cells grown as a monolayer culture are loaded with a membrane-permeant non-fluorescent precursor (e.g., calcein acetoxymethyl ester) which is cleaved inside the cell by esterases, resulting in an impermeable fluorescent form that is trapped intracellularly (Madeira et al., 2014b). Volume changes induced by solute gradients are detected by the change in fluorescence intensity under a fluorescence microscope (Madeira et al., 2013; Madeira et al., 2014b) or alternatively, using a plate-reader that detects the fluorescence of the whole cell layer (Mola et al., 2009; Fenton et al., 2010; Sonntag et al., 2019). While the fluorescence microscope setup allows single cell imaging and analyzing permeability in a single cell mode, the plate reader has the advantage of a high throughput system. However, possible artifacts due to dye leakage or binding to cell membrane represent a significant limitation of this technique (Zelenina and Brismar, 2000; Tradtrantip et al., 2017).

Another fluorescence microscopy method, that does not require dye loading, uses a genetically encoded yellow fluorescent protein (YFP-H148Q-V163S), whose fluorescence is quenched by chloride. The cytoplasmic chloride concentration changes as the cell volume increases or decreases, resulting in altered YFP fluorescence (Galietta et al., 2001; Baumgart et al., 2012; Esteva-Font et al., 2013). The chloride sensor YFP is not affected by leakage artifacts but can be influenced by intracellular pH and anion concentrations, besides is limited time resolution (Galietta et al., 2001; Tradtrantip et al., 2017).

#### Stopped-Flow Spectroscopy

Stopped-flow spectroscopy allows to follow the cell volume changes after a rapidly imposed osmotic or solute gradient. It has been used to quantify water and solute permeabilities in suspended cells, vesicles or liposomes (Madeira et al., 2016; Tradtrantip et al., 2017). To assess aquaglyceroporin function, a cell suspension in isosmotic buffer is rapidly mixed with an equal volume of a hyperosmotic glycerol solution creating an inwardly directed glycerol gradient. The first cell shrinkage due to water efflux caused by the osmotic gradient is followed by water and glycerol influx and subsequent cell reswelling. These cell volume changes can be followed by optical properties of the solution, either light scattering (which is dependent of the cell volume), or fluorescence emission (if cells are loaded with a volume sensitive fluorophore) (Soveral et al., 2007; Madeira et al., 2016). Considering the linear correlation between cell volume and the optical properties of the system, the time course of cell volume changes until osmotic equilibrium is reached allows the quantification of glycerol permeability (Martins et al., 2012; Madeira et al., 2016). Stopped-flow light scattering is susceptible to various artifacts because the light intensity from cells depends not only on cell volume but also on cell shape and refractive index of intracellular and extracellular media. Although fluorescence measurements are relatively insensitive to cell shape, they show similar limitations to the above described for microscopy assays (Tradtrantip et al., 2017; Abir-Awan et al., 2019). Finally, the membrane optical properties can also be influenced by compounds’ treatment, such as AQP modulators.

#### Analysis of Glycerol Permeability and Activation Energy

The evaluation of glycerol permeability (P_gly_) is based on the rate of glycerol movement through membranes caused by a glycerol chemical gradient. When using cell volume measurements (swelling assays, stopped-flow or microscopy), P_gly_ can be calculated by fitting a single exponential equation to the signals obtained after imposing inwardly glycerol gradients, using the relation P_gly_ = k (V_0_/A) (cm s^−1^) where k is the single exponential rate constant and V_0_/A is the initial cell volume to area ratio (Campos et al., 2011; Martins et al., 2012). Alternatively, mathematical models using non-linear equations and numerical integrations can be applied for an accurate determination of P_gly_ (Madeira et al., 2013).

The activation energy (E_a_) of glycerol transport is a measure of the energy barrier needed for glycerol diffusion across the plasma membrane. The E_a_ value is much lower when glycerol permeation occurs *via* aquaglyceroporins than *via* simple diffusion through lipid bilayer, making this parameter extremely valuable to ascertain aquaglyceroporin activity and the proof of concept for the identification of new inhibitors. E_a_ is assessed by performing the permeability assay along a range of temperatures. A weak dependence on the assay temperature indicates that glycerol diffusion *via* the hydrophilic protein pore dominates over the lipid pathway. E_a_ (kcal mol^−1^) can be calculated by the slope of an Arrhenius plot (ln P_gly_ as a function of 1/T) (Madeira et al., 2016).

### Computational Methods

The determination of high-resolution atomic structures of various AQP isoforms was the starting point for the use of computational techniques to investigate AQPs as novel targets in drug discovery. *In silico* approaches have contributed to the understanding of AQPs structure and function, their selectivity for water or other substrates, as well as elucidating structure-activity relationships (Roux and Schulten, 2004; Verkman et al., 2014; Madeira et al., 2016). Until now, the mammalian aquaporins with available high-resolution structures are AQP0 (Harries et al., 2004), AQP1 (Murata et al., 2000), AQP2 (Frick et al., 2014), AQP4 (Ho et al., 2009), AQP5 (Horsefield et al., 2008), AQP7 (de Mare et al., 2020) and AQP10 (Gotfryd et al., 2018). Molecular dynamics (MD) simulations have contributed to explain AQPs function since this technique can follow the rapid water and solute transport through AQPs at an atomic resolution and predict the binding sites of ions and molecules (Roux and Schulten, 2004; Hub et al., 2009). Regarding aquaglyceroporins, the structures of AQP3 and AQP9 have not been solved yet. Therefore, in these cases, three-dimensional homology models were assembled to construct their structures and overcome this limitation allowing to proceed with both classical MD simulations and protein-ligand docking studies (Martins et al., 2012; Wacker et al., 2013). The same homology modeling approach was applied to obtain AQP7 structure before its X-ray structure was solved (Madeira et al., 2014b).

Protein-ligand docking studies have been used as a virtual screening method of potential AQP modulators, being able to predict their binding modes to the protein (Martins et al., 2012; Madeira et al., 2014b; Yadav et al., 2020). Additionally, molecular docking can be combined with molecular dynamics to confirm the proposed inhibitor binding sites, that can also be characterized through density functional theory calculations (Muller et al., 2008; Martins et al., 2012; Verkman et al., 2014; de Almeida et al., 2017).

**FIGURE 3:**
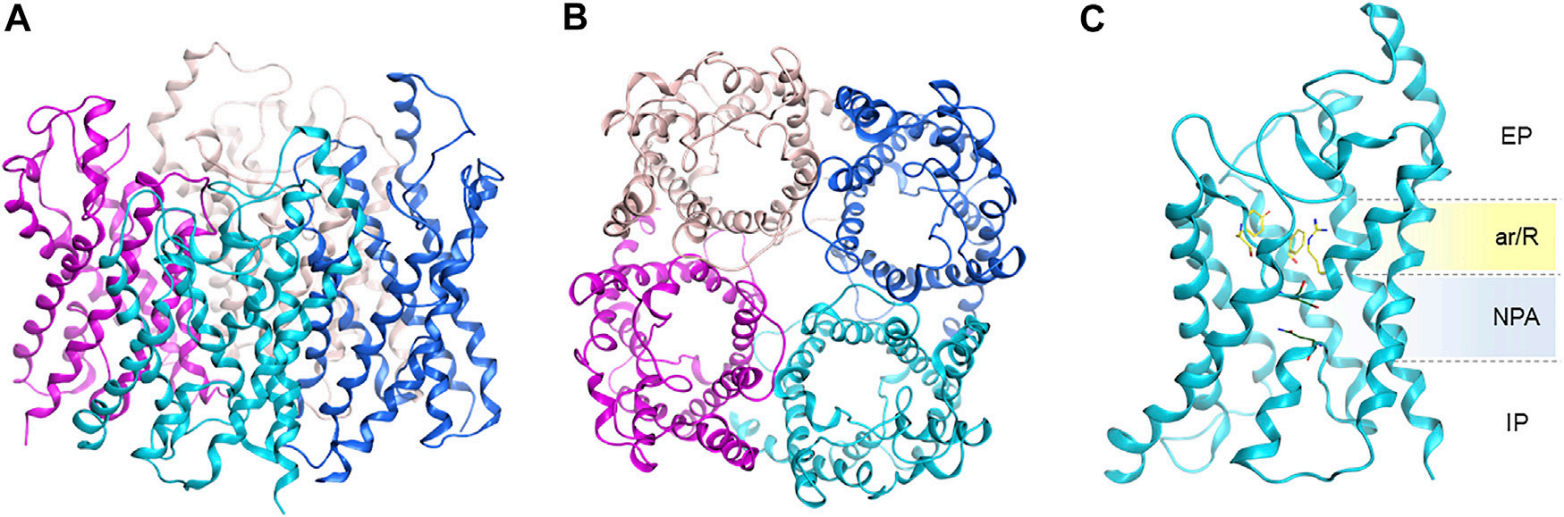
Homology model of human AQP3 tetramer. **(A)** Side, and **(B)** top view; **(C)** AQP3 monomer side view showing position of the ar/R selectivity filter and NPA motifs. EP, extracellular pocket, IP, intracellular pocket. Figure generated using the MOE software (Chemical Computing Group ULC, 2021).

AQPs are assembled as tetramers in the cell membrane (Figure 3). An AQP monomer features an hourglass shaped pore formed by six transmembrane helices, connected by five loops (A through E) and two semi-helices meeting at the center of the channel (Figure 3). Close to the extracellular side, the aromatic/arginine selectivity filter (ar/R SF), the narrowest point of the pore, is responsible for size-exclusion of molecules. Underneath, the pore center is defined by two highly conserved asparagine-proline-alanine (NPA) motifs, contained in the B and E loops and semi-helices. The NPA region has been postulated to be responsible for exclusion of charged solutes through the formation of an electrostatic barrier (Chakrabarti et al., 2004). Specifically, the electrostatic field in this region governs not only the structure and fluctuations of the unprotonated water chain, but also the free energy barrier for an excess proton (Chakrabarti et al., 2004).

MD studies have helped elucidating the role of the selectivity filters and their effects on the permeation of water and other small uncharged solutes (de Groot and Grubmuller, 2001; Janosi and Ceccarelli, 2013). However, while MD calculations can provide free-energies of substrate permeation *via* AQPs by collecting the relevant equilibrium configuration probability distributions, they cannot provide kinetic information of solute conductance. The latter is required for an unbiased mechanistic analysis over a physiologically significant timeframe. In this context, Metadynamics (MetaD) atomistic simulations were used to reconstruct the free-energy profile for substrate permeation from multiple independent runs (Figure 2). MetaD accelerates rare event occurrence along collective variables (CV) and it has recently been successfully applied to elucidate the mechanisms of glycerol and hydrogen peroxide permeation *via* human AQP3 (Wragg et al., 2019; Wragg et al., 2020) as well as of AQP10’s covalent inhibition by small molecules (Pimpao et al., 2021).

## Aquaglyceroporin Modulators With Pharmacological Action

**FIGURE 4:**
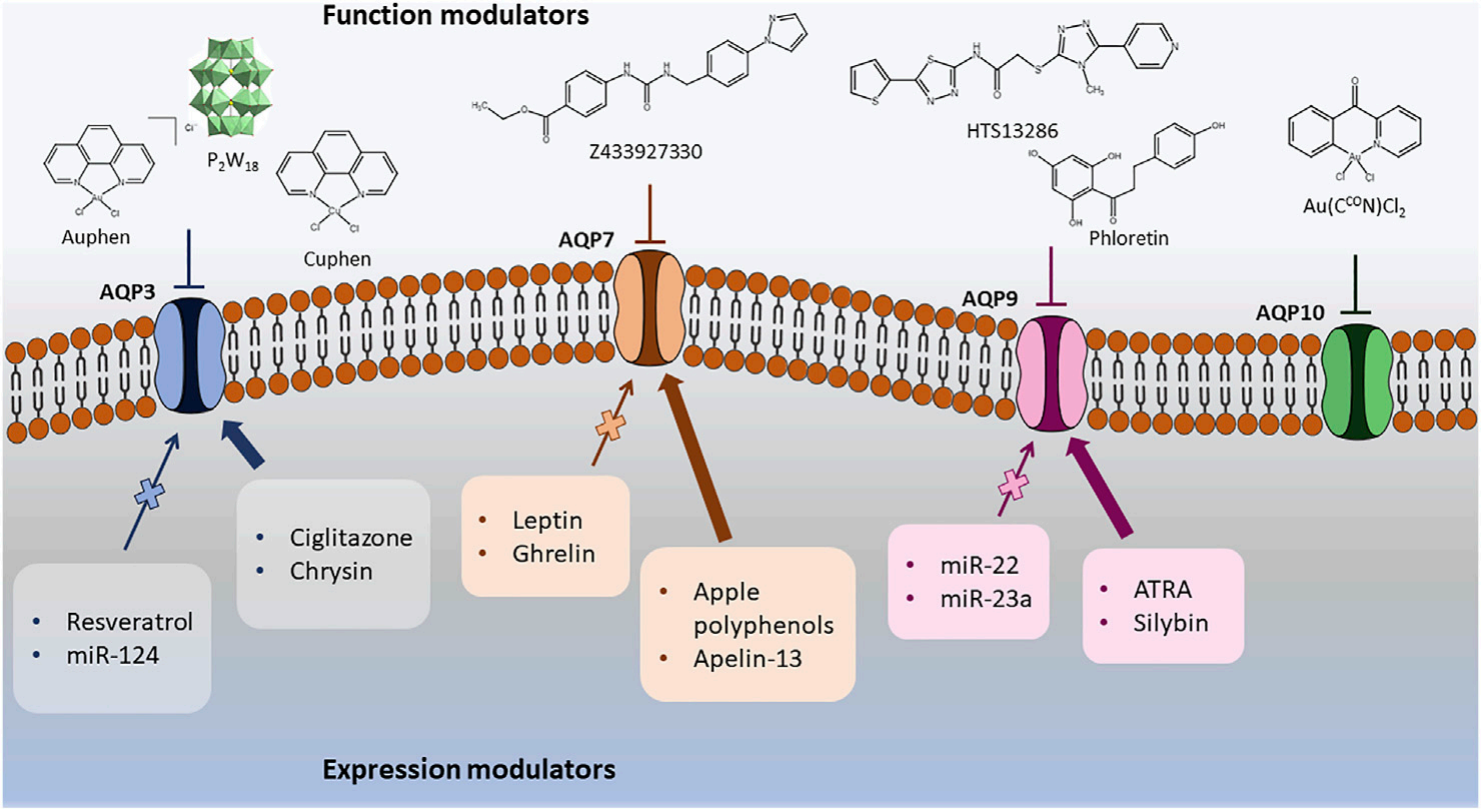
Representative compounds modulators of aquaglyceroporin function or expression.

The discovery of AQPs inhibitors with potential clinical applications has been challenging due to the wide AQP distribution in tissues, the similarity between AQP-isoforms and the structural limitations displayed by the narrow channel pore that is difficult to target, if mere blockage of the substrate permeation by steric effects is envisaged (Verkman et al., 2014; Tradtrantip et al., 2017). However, the pharmacological modulation of aquaglyceroporins has been reported and correlated with potential therapeutic effects in diseases where these proteins are abnormally expressed. Here, we will describe the chemical and biological molecules reported to act as direct inhibitors of aquaglyceroporin function or as modulators of their expression (down or up-regulation), with examples represented in Figure 4. These two types of modulators are included and categorized accordingly in Table 1. In this review, only aquaglyceroporin modulators with reported beneficial effects or claimed to ameliorate the outcome of diseases were considered.

**TABLE 1:** Classes of compounds (transition metal compounds, small molecules, natural compounds and biologicals) modulators of aquaglyceroporin function or expression and related diseases where they may exert a beneficial pharmacological action.

Modulators	AQP	Effect on activity	Effect on expression	Disease/Cell model
1. Transition metal compounds				
Mercury chloride (HgCl_2_)	AQP3	Inhibition	—	Prostate cancer (Ismail et al., 2009)
Nickel chloride (NiCl_2_)	AQP3	Inhibition	—	Human lung epithelial cells Zelenina et al. (2003)
Copper sulfate (CuSO_4_)	AQP3	Inhibition	—	Human lung epithelial cells Zelenina et al. (2004) Human trophoblasts Alejandra et al. (2018)
Auphen	AQP3	Inhibition	—	PC12 cell line Martins et al. (2012) Epidermoid carcinoma Serna et al. (2014) Keratinocytes Yu et al. (2021) Hepatocellular carcinoma Peng et al. (2016) Colonic epithelia cells Thiagarajah et al. (2017)
	AQP7	Inhibition	—	Mouse adipocytes Madeira et al. (2014b)
Au(III) C^CO^N	AQP10	Inhibition	—	Yeast cells expressing hAQP10 Pimpao et al. (2021)
Cuphen	AQP3	Inhibition	—	Skin and colon cancer Nave et al. (2016)
POTs (P_2_W_18_)	AQP3	Inhibition	—	Melanoma Pimpao et al. (2020)
2. Small organic molecules				
Bisacodyl	AQP3	—	Downregulation	Constipation Ikarashi et al. (2011)
Ciglitazone	AQP3	—	Upregulation	Human and mouse keratinocytes Jiang et al. (2011)
SAHA	AQP3	—	Upregulation	Human and mouse keratinocytes Choudhary et al. (2017)
Pioglitazone	AQP7	—	Upregulation	Mouse adipocytes Kishida et al. (2001a)
Rosiglitazone	AQP3	—	Upregulation	Type-2 Diabetes Lee et al. (2005)
	AQP7			
Oleic acid	AQP9	—	Upregulation	Human hepatoma cells Lee et al. (2005)
Wy14643	AQP9	—	Upregulation	Human hepatoma cells Lee et al. (2005)
HTS13286	AQP9	Inhibition	—	Mouse hepatocytes Jelen et al. (2011), Wacker et al. (2013) Rat hepatoma cells Tesse et al. (2021)
DFP00173	AQP3	Inhibition	—	CHO cell line Sonntag et al. (2019)
Z433927330	AQP7	Inhibition	—	CHO cell line Sonntag et al. (2019)
RF03176	AQP9	Inhibition	—	CHO cell line Sonntag et al. (2019)
dbcAMP	AQP9	—	Upregulation	Rat astrocytes Yamamoto et al. (2002) Hepatocellular carcinoma Peng et al. (2016)
3. Natural compounds				
atRA	AQP3	—	Upregulation	UV-induced photoaging Cao et al. (2008)
	AQP9	—	Upregulation	Myeloid leucemia Leung et al. (2007)
Chrysin	AQP3	—	Upregulation	UV-induced photoaging Wu et al. (2011)
Glycolic acid	AQP3	—	Upregulation	UV-induced photoaging Tang et al. (2019)
Resveratrol	AQP3	—	Downregulation	Human keratinocytes Wu et al. (2014)
18β-glycyrrhetinic acid derivative	AQP3	—	Upregulation	Human dermal fibroblasts and keratinocytes Hung et al. (2017)
Curcumin	AQP3	—	Downregulation	Ovarian cancer Ji et al. (2008)
	AQP9	—	Downregulation	Intracerebral hemorrhage Wang et al. (2015)
Daiokanzoto	AQP3	—	Downregulation	Constipation Kon et al. (2018)
RFP	AQP3	—	Downregulation	Constipation Zhu et al. (2019)
Naringenin	AQP3	—	Upregulation	Constipation Yin et al. (2018)
β-patchoulene	AQP3	—	Downregulation	Intestinal mucositis Wu et al. (2021)
Apple polyphenols	AQP7	—	Upregulation	Obesity Boque et al. (2013)
Raspberry ketones	AQP7	—	Upregulation	Obesity Mehanna et al. (2018)
Phloretin	AQP9	Inhibition	—	Mouse macrophages Aharon and Bar-Shavit, (2006), Baldini et al. (2020)
Silybin	AQP9	—	Upregulation	NAFLD (Baldini et al., 2020)
4. Biologicals				
4.1. Hormones				
Insulin	AQP7	—	Downregulation	Type 2 diabetes Kishida et al. (2001b), Kuriyama et al. (2002), Hibuse et al. (2005)
	AQP9	—	Downregulation	Type 2 diabetes Kuriyama et al. (2002)
Estrogen	AQP3	—	Upregulation	Breast cancer Huang et al. (2015)
	AQP7		Upregulation	Fatty liver disease in postmenopausal women Fu et al. (2016)
				Menopausal obesity Jin et al. (2017)
	AQP9		Downregulation	Mouse Sertoli cells Bernardino et al. (2018) Rat epididymis Pastor-Soler et al. (2010)
Testosterone	AQP3	—	Upregulation	Rat epididymis Hermo et al. (2004)
	AQP7	—	Upregulation	Rat uterus Salleh et al. (2015)
	AQP9	—	Upregulation	Rat epididymis Pastor-Soler et al. (2010)
Leptin	AQP3	—	Downregulation	Obesity and NAFLD Rodriguez et al. (2015a)
	AQP7	—	Downregulation	
	AQP9	—	Upregulation	
Ghrelin	AQP7	—	Downregulation	Obesity and type 2 diabetes (Rodriguez et al., 2009) Rat pancreatic β-cells Mendez-Gimenez et al. (2017)
Uroguanylin	AQP3	—	Upregulation	Human visceral adipocytes Rodriguez et al. (2016)
	AQP7	—	Upregulation	
GLP-1	AQP7	—	Downregulation	Rat pancreatic β-cells Mendez-Gimenez et al. (2017)
Dexamethasone	AQP3	—	Upregulation	Human airway epithelial cells Tanaka et al. (1997), Ben et al. (2008)
	AQP7	—	Downregulation	Mouse adipocytes Fasshauer et al. (2003)
Isoproterenol	AQP7	—	Downregulation	Mouse adipocytes Fasshauer et al. (2003)
TNFα	AQP7	—	Downregulation	Mouse adipocytes Fasshauer et al. (2003)
Ambroxol	AQP3	—	Upregulation	Human airway epithelial cells Ben et al. (2008)
Erythropoietin	AQP3	—	Upregulation	Acute renal failure Gong et al. (2004)
4.2. miRNAs				
miR-124	AQP3	—	Downregulation	Hepatocellular carcinoma Chen et al. (2018)
miR-488	AQP3	—	Downregulation	Osteosarcoma Qiu et al. (2018)
miR-874	AQP3	—	Downregulation	Gastric cancer Jiang et al. (2014)
				Intestinal barrier dysfunction Su et al. (2016)
				Pancreatic ductal adenocarcinoma Huang et al. (2017)
				Non-small cell lung cancer Wang et al. (2020)
miR-29a	AQP3	—	Downregulation	Diarrhea-predominant irritable bowel syndrome Chao et al. (2017)
miR-22	AQP9	—	Downregulation	Type 2 diabetes Karolina et al. (2014)
miR-23a	AQP9	—	Downregulation	Type 2 diabetes Karolina et al. (2014)
miR-154-5p	AQP9	—	Downregulation	Chronic constriction injury Wu et al. (2020)
miR-212	AQP9	—	Downregulation	Myocardial infarction Ren and Wang, (2018)
miR-532-5p and miR-532-3p	AQP9	—	Downregulation	Renal cell carcinoma Yamada et al. (2019)
4.3. Peptides				
Apelin-13	AQP7	—	Upregulation	Hypertrophic mouse adipocytes Guo et al. (2014)
4.4. Antibodies				
Anti-AQP3 mAb	AQP3	Inhibition	—	Liver injury Hara-Chikuma et al. (2020)

### Transition Metal Compounds

Mercury compounds such as mercury chloride (HgCl_2_) and p-chloromercuribenzene sulphonate (pCMBS) were the first metal compounds identified as AQP inhibitors and are still used in functional assays despite their associated toxicity and lack of selectivity (Macey and Farmer, 1970; Castle, 2005; Rubenwolf et al., 2012). The mechanism of inhibition has been shown to rely on direct binding of Hg^2+^ to thiol moieties in the AQP channel (Savage and Stroud, 2007; Spinello et al., 2016). Interestingly, mercury chloride was reported to improve the sensitivity of prostate cancer cells to cryotherapy, promoting a higher efficacy of this treatment by inhibiting AQP3 (Ismail et al., 2009). Nickel chloride and copper sulphate were found to inhibit AQP3 in human lung epithelial cells (Zelenina et al., 2003; Zelenina et al., 2004). Later, copper sulphate was used to assess AQP3 role on the extravillous throphoblast (EVT) cell migration and its importance for placentation (Alejandra et al., 2018). Although both nickel chloride and copper sulphate were identified as AQP3 inhibitors, no therapeutic effects have so far been reported for these transition metal compounds.

**FIGURE 5:**
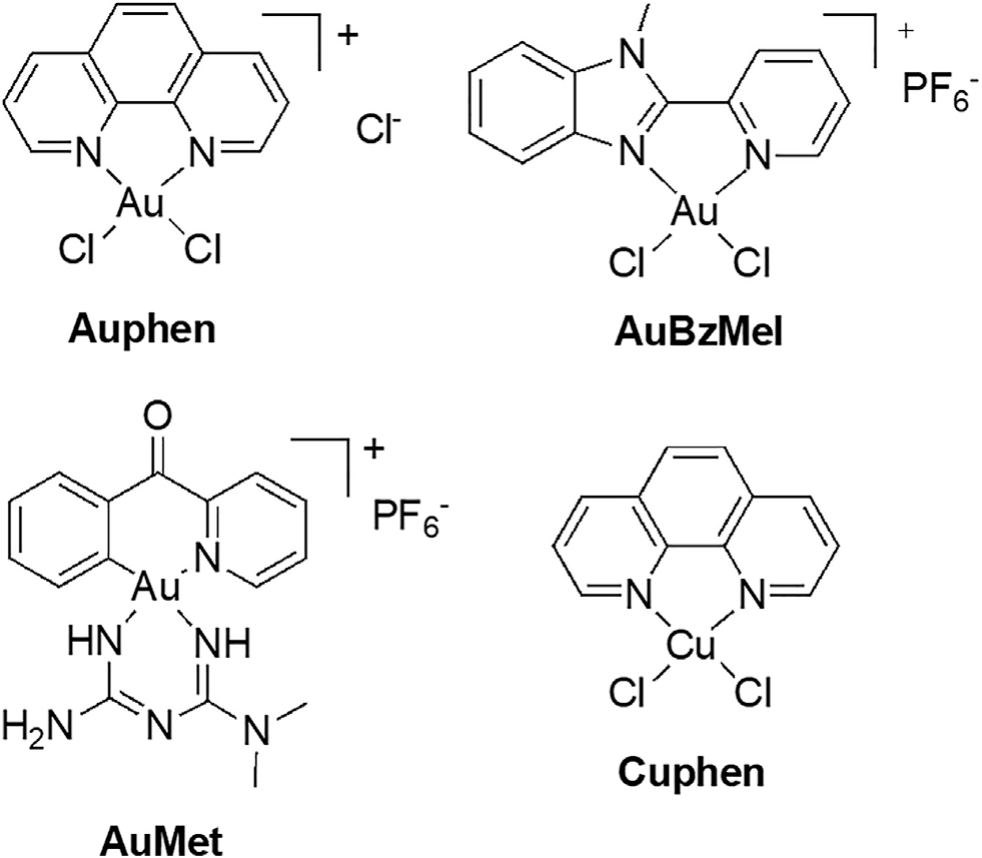
Au(III) and Cu(II) compounds as aquaglyceroporins inhibitors.

AQP3 has been associated with cell proliferation in cancer cells, namely in skin tumors where it is highly expressed (Verkman, 2008b). In recent years, our group has identified gold-based compounds with bidentate N-donor ligands as selective AQP3 inhibitors, with no effects towards orthodox aquaporins (de Almeida et al., 2014). In this series, a gold(III) complex [Au(phen)Cl_2_]Cl (Auphen, phen = 1,10-phenanthroline, Figure 5) (Martins et al., 2012) was found to decrease cell proliferation in AQP3-expressing cells, revealing its potential for the treatment of carcinomas with large AQP3 expression (Serna et al., 2014). The mechanism of inhibition is suggested to occur *via* gold binding to a specific cysteine residue in the AQP3 channel, *via* formation of direct Au-sulfur bonds (Martins et al., 2012; Serna et al., 2014). Further MD studies were conducted for another potent and selective Au(III) inhibitor—the compound [Au(BzMeI)Cl_2_]PF_6_ (AuBzMeI, BzMeI = 1-methyl-2-(pyridin-2-yl)-benzimidazole, Figure 5). The obtained results showed that protein conformational changes upon metal binding to Cys40 in AQP3 are responsible for the observed inhibition of glycerol permeation, and not direct steric blockage of the channel by the metal compound. Overall, in a number of complementary studies (Martins et al., 2013; de Almeida et al., 2017; Graziani et al., 2017), multi-level theoretical approaches have identified three key aspects for AQP3 inhibition by gold compounds: 1) speciation of the gold(III) complex prior protein binding (formation of aquo-complexes), 2) stabilization of non-covalent adducts between the compound organic ligands and the extracellular pore side, and 3) conformational changes induced within the pore by the coordinative binding of Au(III) ions leading to pore closure.

Auphen was also shown to inhibit AQP7 in adipocytes (Madeira et al., 2013; Madeira et al., 2014b) and was used to unveil a role of AQP3 in keratinocytes autophagy (Yu et al., 2021), to suppress growth of hepatocellular carcinoma by downregulating AQP3 (Peng et al., 2016), and to impair cell migration *via* AQP3 blockage in colonic epithelia (Thiagarajah et al., 2017). Additionally, AQP3 inhibition by Auphen in monocytic THP-1 cells was shown to reduce IL-1β release and pyroptosis by preventing NLRP3 inflammasome activation induced by reswelling, nigericin and ATP (da Silva et al., 2020b).

**FIGURE 6:**
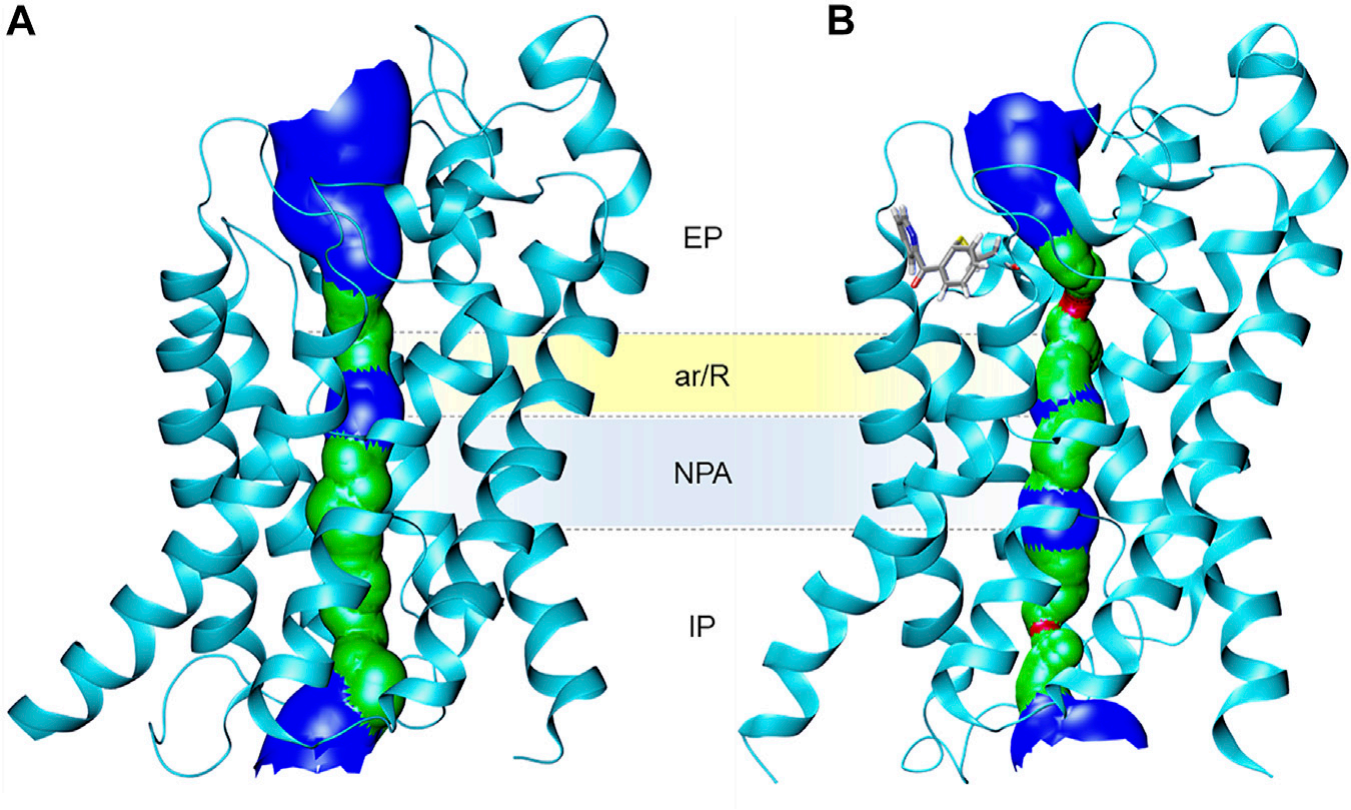
Ribbon representation of **(A)** the human AQP10 monomer and **(B)** the AQP10-C^N adduct with modified Cys209, showing the effects of pore size as a 3D representation (based on VDW radius: red = smaller than single H_2_O, green = single H_2_O, blue = larger than single H_2_O). Cys209-C^N fragment is shown in stick representation with atoms coloured by atom type (red = oxygen, blue = nitrogen, grey = carbon, yellow = sulfur). Figure generated using HOLE (Smart et al., 1996) and VMD software (Humphrey et al., 1996).

Recently, new organogold derivatives of general formula [Au(C^N)Cl_2_] (C^N = cyclometalated ligand), being endowed with increased stability in aqueous environment relative to the benchmark inhibitor Auphen (Casini et al., 2008; Bertrand and Casini, 2014), were shown to inhibit human AQP10 in a yeast model (Pimpao et al., 2021). Some of the tested gold complexes have been reported to inhibit cancer cells proliferation *in vitro* (Bertrand et al., 2015) and *in vivo* (Babak et al., 2021). Thus, the Au(III) C^N complex featuring a metformin ancillary ligand (AuMet, Figure 5) was 6000-fold more cytotoxic compared to uncoordinated metformin and significantly reduced tumor burden in mice with aggressive breast cancers. Noteworthy, the organogold compounds feature a peculiar reactivity with cysteine residues, whereby, following gold adduct formation, the reaction proceeds towards irreversible cysteine arylation, whereby coupling the C^N ligand (2-benzoylpyridine) to the side-chain thiol occurs *via* reductive elimination at the Au(III) centre. MetaD provided initial mechanistic insights on the effects of the inhibitors’ binding to AQP10, showing that cysteine arylation reduces glycerol conductance (Pimpao et al., 2021). In details, upon covalent binding of the C^N ligand to Cys209 in AQP10, the pathway of glycerol permeation was significantly altered due to an overall shrinkage of the pore, while water flux was only minimally affected (Figure 6).

The copper(II) analogue of Auphen, [Cu(phen)Cl_2_] (Cuphen, Figure 5) was also observed to inhibit AQP3, although less potently than Auphen, and to reduce glycerol permeation in AQP3-expressing melanoma (Nave et al., 2016; Pinho et al., 2019) and colon cancer cells (Pinho et al., 2021), leading to anti-proliferative effects and cell migration impairment. Of note, when formulated in nanoliposomes targeting solid tumors, Cuphen-loaded nanoliposomes were able to reduce tumor growth in mice bearing melanoma (Pinho et al., 2019) and colon cancer (Pinho et al., 2021). Finally, the ability of polyoxotungstates (POTs), in particular P_2_W_18_, to inhibit AQP3 and impair melanoma cell migration was reported, suggesting that the well-known anticancer properties of these compounds may in part be due to the blockage of AQP3-mediated permeability (Pimpao et al., 2020). Altogether, these studies unveil the potential of metallodrugs as anticancer agents against tumors where AQP3 is highly expressed.

### Small Organic Molecules

Small molecules with low molecular weight (<900 Da) have been reported to modulate aquaglyceroporins expression and function (Figure 4), exerting a beneficial influence on disease outcomes. For instance, bisacodyl, a laxative widely used to treat constipation, was reported to decrease AQP3 protein expression in rat colon through activation of macrophages and increased PGE2 secretion and was closely associated with an increase in fecal water content, suggesting that bisacodyl may exert a laxative effect due to AQP3 modulation (Ikarashi et al., 2011). Ciglitazone, a peroxisome proliferator-activated receptor gamma (PPARγ) activator, and Suberanilohydroxamic acid (SAHA), a histone deacetylase (HDAC) inhibitor, were reported to increase AQP3 mRNA and protein levels in human and mouse keratinocytes, leading to an increase in glycerol uptake. These studies indicate the potential of ciglitazone and SAHA as therapeutic agents in skin disorders (Jiang et al., 2011; Choudhary et al., 2017).

The promoters of the genes encoding AQP3, AQP7 and AQP9 exhibit putative peroxisome proliferator response elements (PPRE), suggesting that PPAR can influence AQP expression (Kishida et al., 2001a; Patsouris et al., 2004; Jiang et al., 2011). In fact, pioglitazone, a PPARγ agonist, activates PPARγ and leads to augmented AQP7 mRNA levels and promoter activity in mice adipocytes (Kishida et al., 2001a). Rosiglitazone was also shown to increase AQP7 mRNA levels in the mesenteric fat and induce glycerol kinase mRNA expression in both fat depots on OLETF rats, a type 2 diabetes model. These findings indicate that rosiglitazone can regulate AQP7 and glycerol kinase expression in adipose tissue, playing an important role in glycerol metabolic pathway. AQP9 and glycerol kinase mRNA levels were not affected with rosiglitazone administration to OLETF rats (Lee et al., 2005). However, AQP9 protein expression was upregulated after treatment with oleic acid and Wy14643 in cultured human hepatoma cells. These results suggest that fatty acids can promote glycerol availability in the liver through AQP9 upregulation. AQP3 mRNA levels were also found to be increased in the outer medulla of OLETF rat kidney after rosiglitazone treatment, which could be related to the fluid retention caused by this compound (Lee et al., 2005).

A series of small molecules were tested as aquaglyceroporin inhibitors highlighting HTS13286 as AQP9 inhibitor and with potential to reduce intracellular glycerol accumulation and impair gluconeogenesis in murine liver (Jelen et al., 2011; Wacker et al., 2013). A recent study reported the ability of HTS13286 to prevent LPS-induced systemic inflammation *via* AQP9 blockage in rat hepatoma cells (Tesse et al., 2021). However, due to its low water solubility, HTS13286 may not be appropriate for *in vivo* experiments, with structural analogues with higher potency and increased water solubility being suggested as promising molecules to control gluconeogenesis in type-2 diabetes (Jelen et al., 2011; Wacker et al., 2013). Recently, the compounds DFP00173 and Z433927330 were shown as potent AQP3 and AQP7 inhibitors, and RF03176 was able to significantly impair H_2_O_2_ permeability in AQP9-expressing CHO cells. These three compounds exert their inhibitory effect by binding to AQP cytoplasmic entrance (Sonntag et al., 2019).

Dibutyryl cyclic adenosine monophosphate (dbcAMP), a cell-permeable synthetic analogue of cAMP, was found to upregulate AQP9 mRNA and protein expression in rat astrocytes. The effects of dbcAMP on AQP9 mRNA levels were reverted by pre-treating astrocytes with protein kinase A (PKA) inhibitors, suggesting that PKA is involved in the regulation of AQP9 expression (Yamamoto et al., 2002). The increase on AQP9 mRNA and protein levels through dbcAMP modulation was also confirmed in a mice model of HCC, resulting in tumor growth suppression with decrease in tumor size (Peng et al., 2016).

### Natural Compounds

Natural compounds are bioactive products derived from natural sources, usually plants, that are known for their beneficial effects in disease prevention and in reducing the risk of chronic diseases including cardiovascular diseases and cancer (Maraldi et al., 2014; Tesse et al., 2018). These bioactive compounds can exert therapeutic effects on AQPs by influencing their expression and/or function (Tesse et al., 2018; Portincasa and Calamita, 2019).

UV radiation causes human skin photoaging, characterized by dehydration and delayed wound healing, and was found to downregulate AQP3 protein expression in human keratinocytes. All-trans retinoic acid (atRA), chrysin and glycolic acid were found to revert the UV-induced AQP3 downregulation in human keratinocytes, revealing their protective role against UV-induced photoaging (Cao et al., 2008; Wu et al., 2011; Tang et al., 2019). Interestingly, both atRA and chrysin exert their effect through activation of ERK signaling pathway and involvement on redox signaling and oxidative stress (Cao et al., 2008; Wu et al., 2011). atRA can also induce AQP9 mRNA and protein expression resulting in increased As_2_O_3_ uptake in myeloid leukemia cells and, consequently, augmented arsenic sensitivity (Leung et al., 2007). In addition, resveratrol was shown to downregulate AQP3 mRNA and protein expression in a SIRT1/ARNT/ERK dependent manner in normal human epidermal keratinocytes, resulting in the impairment of cell proliferation, which was also observed in AQP3-knocked down cells (Wu et al., 2014). Glycyrrhizin is a bioactive compound of licorice that can be hydrolyzed to 18β-glycyrrhetinic acid which has immunomodulatory, anti-inflammatory, anti-carcinogenic and antioxidant properties. Interestingly, a 18β-glycyrrhetinic acid derivative was found to increase AQP3 mRNA and protein expression and enhance both proliferation and migration in human dermal fibroblasts and keratinocytes, revealing its potential usage in skin diseases with impaired wound healing and skin lesions (Hung et al., 2017).

Curcumin, a non-flavonoid polyphenol known for its anticancer properties, suppresses EGF-induced AQP3 protein expression upregulation and decreases cell migration in cultured human ovarian cancer cells, with inhibitory effect on EGF-induced EGFR, PI3K/AKT, and MEK/ERK activation. The same effect was observed with AQP3 knockdown or by treating cells with AQP3 inhibitors (NiCl_2_ and CuSO_4_) (Ji et al., 2008). This same compound was reported to attenuate brain edema and neurological deficits in mice with intracerebral hemorrhage (ICH), through the reduction of AQP9 mRNA and protein expression *via* reduced activation of NF-kB pathway, showing therapeutic potential as a treatment for this disease (Wang et al., 2015). Daiokanzoto administration in rats, a combination of sennoside A and glycyrrhizin, and treatment with a purified resin glycoside fraction from Pharbitis Semen (RFP) on human colonic epithelial cells suppresses AQP3 protein expression accompanied with an increase in fecal water content, indicating that both compounds can exert a laxative effect through AQP3 modulation and could be used for chronic constipation treatment (Kon et al., 2018; Zhu et al., 2019). Surprisingly, in mice colon with loperamide-induced constipation, the flavanone naringenin was found to upregulate AQP3 mRNA and protein expression, as opposed to the effect of daiokanzoto and RFP, but also causing an increase in fecal water content (Yin et al., 2018). Recently, β-patchoulene, the main active ingredient extracted from patchouli oil, was reported to decrease AQP3 abnormal protein expression in intestinal mucositis-induced rats through inactivation of cAMP/PKA/CREB signaling pathway, restoring water transport and attenuating diarrhea symptoms (Wu et al., 2021).

Raspberry ketones and apple polyphenols were reported to upregulate AQP7 mRNA expression in diet-induced obese rats (Boque et al., 2013; Fiorentini et al., 2015; Mehanna et al., 2018). Moreover, apple polyphenols could revert the AQP7 promoter hypomethylation caused by high-fat sucrose diet. The observed increase in glycerol release in *ex vivo* lipolysis experiments and the reduction in adipocyte size, could be explained by AQP7 upregulation for its crucial role in adipocyte glycerol permeability and control of triglyceride accumulation (Boque et al., 2013; Fiorentini et al., 2015). Phloretin, a chalcone abundant in apples, was identified as a non-specific AQP9 inhibitor that can reduce the size of bone-resorbing cells, the osteoclasts, with inhibition of osteoclast differentiation, a biological process mediated by AQP9 (Tsukaguchi et al., 1999; Aharon and Bar-Shavit, 2006). Recently, silybin, the major bioactive component of milk thistle, was found to upregulate AQP9 mRNA and protein expression in hepatic steatosis with increase in glycerol uptake, preventing triglycerides accumulation and liver dysfunction with decreased fat-induced autophagy. These novel findings suggest a therapeutic effect towards hepatic steatosis in NAFLD (Baldini et al., 2020).

### Biologicals

Hormones, microRNAs (miRNAs) and peptides, called “biologicals,” are naturally occurring molecules with therapeutic properties which have also been described as modulators of aquaglyceroporins’ expression, as described below.

#### Hormones

Modulation of aquaglyceroporins’ expression by hormones has been well documented in the literature. The tight regulation of AQP7 expression in insulin-sensitive tissues and the modulation of AQPs by gonadal steroids in female and male reproductive systems, are examples of the important roles played by these protein channels in metabolism and reproductive health (Wawrzkiewicz-Jalowiecka et al., 2021). Insulin is known to repress AQP7 transcription in mice adipocytes (Kishida et al., 2001b), with AQP7 KO mice showing insulin resistance and triglycerides accumulation in adipose tissue (Hibuse et al., 2005). In fact, both AQP7 and AQP9 expression are inversely modulated by insulin, leading to increased AQP7 and AQP9 expression during fasting and decreased expression during feeding (Kuriyama et al., 2002; Galli et al., 2021).

The estrogen 17β-estradiol was reported to decrease lipogenesis by upregulating the mRNA and protein expression of AQP7 in mice, being related to the amelioration of ovariectomy-induced hepatic steatosis that is characterized by a decrease in estrogen secretion. These findings suggest that estrogen may play a key role on hepatocyte protection from steatosis through increase of AQP7 expression (Fu et al., 2016). The therapeutic effect of this hormone in menopause-related diseases was once again associated with modulation of AQP7 expression. 17β-estradiol supplement reversed the AQP7 mRNA and protein expression downregulation and glycerol kinase upregulation in visceral adipose tissue in ovariectomized rats, with no effect on the subcutaneous adipose tissue, suggesting its therapeutic potential to prevent the development of obesity in postmenopausal women (Jin et al., 2017). Moreover, high levels of estrogen resulted in AQP9 downregulation in Sertoli cells (Bernardino et al., 2018; Carrageta et al., 2020) which was also observed in male rat epididymis efferent ducts, confirming AQP9 estrogen sensitivity. However, this effect could be reverted with testosterone administration (Pastor-Soler et al., 2010). Additionally, in orchiectomized rat epididymis, AQP3 expression was suppressed and was partially recovered by testosterone treatment (Hermo et al., 2004). Testosterone also induced AQP7 upregulation in the uterus of ovariectomized rats, leading to decreased uterine fluid volume (Salleh et al., 2015). In addition, AQP3 upregulation by estrogen in estrogen receptor-positive breast cancer and by both estrogen and progesterone in endometrial cells, affected cancer cell migration and invasion through reorganization of actin cytoskeleton and EMT (Huang et al., 2015; Cui et al., 2018).

Leptin is an endocrine hormone with a well-known role in body energy homeostasis. Leptin deficiency was associated with obesity and NAFLD showing increased AQP3 and AQP7 expression levels in white adipose tissue with no changes in AQP9 expression. Leptin chronic administration to leptin deficient ob/ob mice decreased AQP3 and AQP7 mRNA and protein levels in adipocytes while hepatic AQP9 was increased, unveiling a potential strategy to control lipid accumulation in adipose tissue and liver while maintaining normal glycemia in obese individuals (Rodriguez et al., 2009). Leptin treatment reduced the transcription of the lipogenic factor PPARγ, and AQP7 induced expression with rosiglitazone administration, a well-know PPARγ agonist, in murine adipocytes. However, leptin treatment in AML12 hepatocytes was reported to increase basal and rosiglitazone-induced AQP9 transcription with slight downregulation of PPARγ mRNA expression (Rodriguez et al., 2015a).

The gut hormone ghrelin downregulates AQP7 protein expression, responsible for lipolysis-derived glycerol efflux in human visceral adipose tissue and plays a role in the excessive fat accumulation in obese individuals (Rodriguez et al., 2009). Both forms of acylated and deacyl ghrelin were reported to downregulate AQP7 mRNA and protein levels in rat pancreatic β-cells together with increased triglycerides content. Another gut hormone, uroguanylin, stimulates lipolysis in human adipocytes and upregulates in parallel genes involved in glycerol (AQP3 and AQP7) and free fatty acids (FATP1 and CD36) efflux (Rodriguez et al., 2016). Moreover, glucagon-like-peptide 1 (GLP-1) also downregulates AQP7 in the same pancreatic cells, with an increase in insulin release. AQP7 reduced levels by ghrelin and GLP-1 can promote intracellular glycerol accumulation, a substrate for triglycerides biosynthesis and insulin synthesis and secretion in β-cells (Mendez-Gimenez et al., 2017).

Dexamethasone and ambroxol were identified as AQP3 modulators, capable of upregulating its mRNA and protein expression with increase in water permeability in human airway epithelial cells. Considering the role of AQP3-mediated water transport in the airways, increasing AQP3 expression through modulation with corticosteroids as dexamethasone and ambroxol could exert a therapeutic effect on pulmonary diseases with airway hypersecretion through clearance of pulmonary fluid (Tanaka et al., 1997; Ben et al., 2008). Moreover, AQP7 mRNA levels were downregulated by treating 3T3-L1 adipocytes with isoproterenol, tumor necrosis factor alpha (TNFα) and dexamethasone (Fasshauer et al., 2003).

Erythropoietin is a glycoprotein hormone, naturally produced by the peritubular cells of the kidney. Erythropoietin was found to prevent the ischemia-induced downregulation of AQP3 protein expression in rats with acute renal failure, improving their urinary concentrating capability. Moreover, the combination of erythropoietin with α-melanocyte-stimulating hormone boosted this effect, being proposed to prevent ischemia/reperfusion injuries through upregulation of renal AQPs (Gong et al., 2004).

#### MicroRNAs

MicroRNAs (miRNAs) are small, single-stranded non-coding RNAs that have emerged as important post-transcriptional regulators of gene expression (Kan et al., 2012; Christopher et al., 2016; Gomes et al., 2018). miRNAs have been identified as endogenous modulators of AQPs, indicating their potential as a therapeutic approach in AQP-related disorders (Gomes et al., 2018).

Overexpression of AQP3 has been observed in different types of cancer such as lung, pancreas, skin, cornea and colon cancer, where it is involved in cell migration, invasion, proliferation, and EMT (Verkman et al., 2008; Marlar et al., 2017). Accordingly, most of the miRNAs described until now as AQP3 modulators exert their beneficial effects in cancer cells by attenuating their growth and metastasis throughout the body. For instance, the overexpression of miR-124 was reported to downregulate AQP3 mRNA and protein expression, leading to the inhibition of proliferation and migration of HCC cells. Moreover, circular RNA HIPK3 (circHIPK3) was identified as a miR-124 sponge, thereby regulating the proliferation and migration of the HCC cells *via* miR-124-AQP3 axis through miR-124 downregulation and AQP3 upregulation. When circHIPK3 was silenced, both AQP3 mRNA and protein levels were reduced and miR-124 was upregulated, resulting in reduced HCC cells proliferation *in vitro* and suppressed tumor growth *in vivo* (Chen et al., 2018).

In osteosarcoma (OS) tissues and cells, miR-488 was found to be downregulated while AQP3 was markedly increased. Accordingly, the introduction of miR-488 leads to a suppression of the proliferation, invasion and EMT of OS cells through the downregulation of AQP3 mRNA and protein expression by binding to AQP3 3′UTR, supporting the idea that both miR-488 and AQP3 could have prognostic value in OS (Qiu et al., 2018). miR-874 has been reported to target AQP3 in gastric cancer (GC) (Jiang et al., 2014), pancreatic ductal adenocarcinoma (PDAC) (Huang et al., 2017) and non-small cell lung cancer (NSCLC) (Wang et al., 2020). In GC tissues and cells, mir-874 can inhibit cell proliferation, migration and invasion through reduction in AQP3 protein expression (Jiang et al., 2014). Moreover, a mouse model of intestinal ischaemia reperfusion injury showed increased miR-874 levels and decreased AQP3 protein levels, an effect that was similarly observed in Caco-2 cells under hypoxia, where miR-874 induced paracellular permeability by inhibiting AQP3 expression contributing to the impairment of intestinal barrier integrity (Su et al., 2016). This same effect was reported for miR-29a in diarrhea-predominant irritable bowel syndrome rat models (Chao et al., 2017). Overexpression of long non-coding RNA (lncRNA) H19, a miR-874 sponge, could revert the miR-874 induced effects and reduce paracellular permeability, suggesting its potential role in reestablishing the intestinal barrier function (Su et al., 2016). In PDAC cells, miR-874 downregulates AQP3 mRNA and protein expression that in turn can lead to inhibition of cell growth and promotion of cell apoptosis through mTOR signaling pathway modulation (Huang et al., 2017). Regarding NSCLC, the same miRNA was reported to inhibit proliferation, invasion and EMT *in vitro* through downregulation of AQP3 mRNA and protein levels with inhibition of PI3K/AKT signaling pathway and was also found to suppress cell growth *in vivo* (Wang et al., 2020).

Further studies identified miR-22 and miR-23a as AQP9 modulators by downregulating its mRNA and protein expression in cultured human hepatocytes and in diabetic rats livers, suggesting that their role in the regulation of glycerol entrance in these cells could be beneficial for the control of glycerol-based gluconeogenesis that is often augmented in diabetes (Karolina et al., 2014). AQP9 was also reported to be modulated by miR-154-5p, which in turn is regulated by lncRNA MALAT-1 in chronic constriction injury (CCI) rats. The downregulation of this lncRNA was found to attenuate neuropathic pain in rats by binding to miR-154-5p that in turn can inhibit AQP9 mRNA and protein expression, unraveling the potential of MALAT-1/miR-154-5p/AQP9 axis as a therapeutic target for neuropathic pain (Wu et al., 2020). Additionally, in rats with myocardial infarction (MI), miR-212 overexpression was suggested to downregulate AQP9 mRNA and protein expression through PI3K/AKT signaling pathway activation, thereby exerting its therapeutic effect by reducing cardiomyocytes apoptosis, promoting vascular regeneration and attenuating ventricular remodeling (Ren and Wang, 2018). Moreover, recent findings showed AQP9 as a target for both miR-532-5p and miR-532-3p in renal cell carcinoma (RCC) cells, being its aberrant expression intimately associated with poor prognosis of RCC patients. However, when both miR-532-5p and miR-532-5p were overexpressed in RCC cells resulting in reduction of AQP9 mRNA and protein levels, cell proliferation, migration and invasion were attenuated. Similar effect was attained with AQP9 knockdown with decrease in cancer aggressiveness, suggesting AQP9 as a promising therapeutic target for RCC (Yamada et al., 2019).

#### Peptides

Peptides have been receiving great attention for their ability to establish specific and high-affinity interactions with endogenous receptors (Martinez-Villaluenga and Hernandez-Ledesma, 2020). In fact, bioactive peptides can act as hormones, neurotransmitters, and antimicrobial agents *in vivo*, with reported beneficial effects on blood pressure and lipid metabolism as well as anticancer, immunomodulatory, analgesic, antioxidant and anti-inflammatory activity (Tinoco and Saghatelian, 2011; Cicero et al., 2017). Considering their health-promoting effects, peptides have been investigated for their potential role as aquaporin modulators.

Apelin-13 is a small peptide found in the adipose tissue that is thought to be implicated on energy metabolism and insulin sensitivity, being also associated with obesity. This peptide was shown to decrease triglycerides accumulation in hypertrophic adipocytes *in vitro* through AQP7 mRNA and protein upregulation *via* PI3K signaling pathway, revealing that apelin-13 is a promising starting point to develop novel treatments against obesity and related health problems (Guo et al., 2014).

#### Antibodies

Recently, Hara-Chikuma et al. (2020) developed an anti-AQP3 monoclonal antibody that targets an extracellular epitope of AQP3 in macrophages. The administration of this anti-AQP3 monoclonal antibody in a liver injury mice model resulted in the suppression of inflammation and liver injury through the inhibition of AQP3-mediated H_2_O_2_ transport, NF-κB signaling and macrophage activation. This study emerges as a starting point for antibodies’ production to specifically target AQPs and develop directed therapies against AQP-associated disorders.

## Conclusion

Aquaglyceroporin modulators have gained much attention in recent years and their development is currently considered a promising strategy for treatment of AQP-associated pathologies. The vast array of compounds reported to affect aquaglyceroporins expression or function has been growing, as an attempt to validate their pharmacological activity for human diseases. However, the application of aquaglyceroporin modulators in clinical trials is still far to be implemented, possibly due the lack of selective and potent modulators able to be administered *in vivo*.

Different classes of molecules, from metal compounds and small molecules to natural compounds and biologicals, have been reported to effectively target aquaglyceroporins, either modifying pore permeability or protein expression level. Yet, their mechanisms of action are obscure and the proof of concept for their pharmacological activity in most animal models of disease is still unmet. While advanced atomistic simulations can provide valuable information on the mechanisms of selectivity and substrate transport *via* AQPs, as well as insights into the mechanisms of their inhibition by different substances, further studies are needed to improve the efficiency and selectivity of modulators, avoiding cross-reactivity with other targets and potential side effects. Given the broad range of diseases where aquaglyceroporins play a role, the design and development of new potent and selective modulators may pave the way to the discovery of innovative therapeutics.
